# Direct view on the phase evolution in individual LiFePO_4_ nanoparticles during Li-ion battery cycling

**DOI:** 10.1038/ncomms9333

**Published:** 2015-09-23

**Authors:** Xiaoyu Zhang, Martijn van Hulzen, Deepak P. Singh, Alex Brownrigg, Jonathan P. Wright, Niels H. van Dijk, Marnix Wagemaker

**Affiliations:** 1 Department of Radiation Science and Technology, Delft University of Technology, Mekelweg 15, 2629JB Delft, The Netherlands; 2 European Synchrotron Radiation Facility, 6 rue Jules Horowitz, BP 220, 38043 Grenoble Cedex, France

## Abstract

Phase transitions in Li-ion electrode materials during (dis)charge are decisive for battery performance, limiting high-rate capabilities and playing a crucial role in the cycle life of Li-ion batteries. However, the difficulty to probe the phase nucleation and growth in individual grains is hindering fundamental understanding and progress. Here we use synchrotron microbeam diffraction to disclose the cycling rate-dependent phase transition mechanism within individual particles of LiFePO_4_, a key Li-ion electrode material. At low (dis)charge rates well-defined nanometer thin plate-shaped domains co-exist and transform much slower and concurrent as compared with the commonly assumed mosaic transformation mechanism. As the (dis)charge rate increases phase boundaries become diffuse speeding up the transformation rates of individual grains. Direct observation of the transformation of individual grains reveals that local current densities significantly differ from what has previously been assumed, giving new insights in the working of Li-ion battery electrodes and their potential improvements.

## Abstract (web summary)

Understanding phase transitions in electrodes under realistic conditions is important for future battery design. Here, the authors use synchrotron micro-beam diffraction to reveal the phase transition mechanism within individual particles of LiFePO_4_, revealing a cycling rate transformation mechanism.

## Graphical abstract

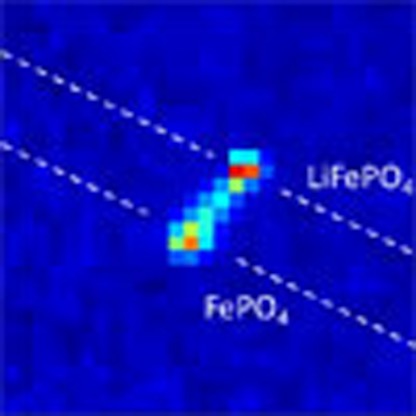

Electrochemical energy storage in Li-ion batteries has enabled the development of today’s portable electronics and is expected to be the key technology for electrical vehicles, as well as for lifting the difference between supply and demand of renewable energy sources. The working of Li-ion batteries is based on the reversible insertion and extraction of Li-ions in the crystal structure of the positive and negative electrode materials resulting in highly efficient and high-density energy storage. Large volume changes, associated with phase transitions in the electrode material, typically result in poor cycle performance by mechanical failure. In addition, the formation of interfaces, that occur as a result of the phase transformation, are associated with a poor rate performance, whereas solid solution reactions typically lead to high (dis)charge rates.

Generally, the overall phase transformation behaviour of Li-ion battery electrode materials is well known. For instance the intensively studied model system and important LiFePO_4_ (refs 1, 2) -positive electrode material combines a first-order phase transition with surprisingly high-rate performance and cycle life^3^,^4^,^5^,^6^,^7^,^8^. The high-rate performance has recently been explained by the possibility to bypass the two-phase reaction through a solid solution reaction at high rates^9^,^10^ as predicted by modelling^11^,^12^,^13^.

However, even for well-studied electrode materials such as LiFePO_4_ it is unknown how the transformation proceeds in individual grains and how it is distributed over the many particles present in an electrode. This is crucial information as the local conditions govern the Li-ion battery cycle life and rate performance, and are key towards the interpretation of easy to measure macroscopic electrochemical parameters. For example, the transformation rate of individual grains defines the maximum (dis)charge current that can be achieved by Li-ion battery electrodes. In addition, it determines what fraction of the electrode is concurrently transforming, which in case of a small active fraction carries a large fraction of the overall current^14^ leading to local hotspots that are detrimental for the cycle life^15^,^16^. Large local current densities in LiFePO_4_ are predicted based on the particle-by-particle, or mosaic, transformation^14^,^17^,^18^,^19^,^20^,^21^,^22^,^23^,^24^,^25^ mechanism, avoiding phase coexistence within individual crystallites. The formulation of the particle-by-particle transformation is based on the absence of phase coexistence within individual crystallites as first suggested by *ex-situ* powder X-ray diffraction^17^ and subsequently supported by *ex-situ* electron^18^,^19^ and X-ray microscopy^14^,^20^. However, the particle-by-particle (mosaic) transformation at low rates is not undisputed, as *in-situ* neutron diffraction^26^ supports a more concurrent transformation in LiFePO_4_. The associated phase coexistence has also been observed with *ex-situ*^27^,^28^,^29^ TEM, recent *in-situ* TEM^30^,^31^ and IR spectroscopy^32^, challenging the particle-by-particle transformation mechanism.

The restricted knowledge of the local phase transition behaviour in LiFePO_4_, and of insertion electrodes in general, is at least partially the consequence of the absence of experimental techniques with sufficient space and time resolution to monitor the nucleation and growth mechanism within individual grains and their distribution over the electrode grains under realistic (dis)charge conditions. This hinders direct insight in crucial battery properties, such as the cycling rate and cycle life, which is paramount for the development of electrodes with improved rate and cycle performance. In this study, for the first time operando microbeam X-ray diffraction is employed to monitor the transformation of many individual LiFePO_4_ particles inside a Li-ion battery electrode from low to high cycling rates. This reveals the cycling rate-dependent phase transition mechanism within individual electrode grains. In contrast to the in LiFePO_4_ commonly assumed fast particle-by-particle or mosaic transformation, we observe at low cycling rates that individual electrode grains transform very slowly and concurrently via coexisting platelet-shaped phase domains. At fast cycling the platelet-shaped domains make room for diffuse interphases proving a distribution of instable intermediate phases within a single electrode grain.

## Results

### Monitoring the phase transition in individual grains

**Figure 1:**
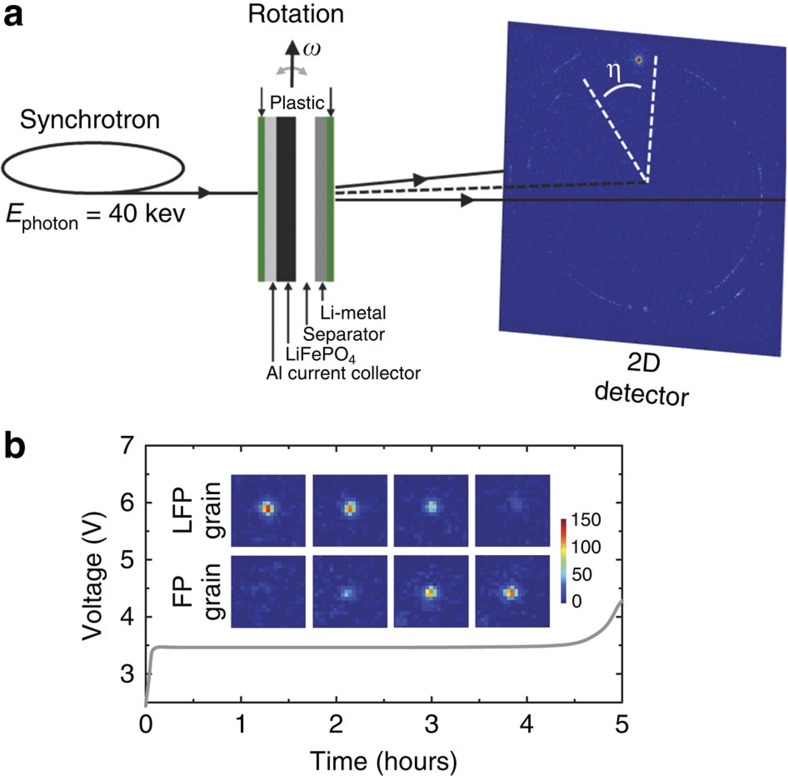
Schematic representation of the *in situ* synchrotron X-ray diffraction experiment. (**a**) Schematic representation of experimental setup. During the exposure the sample was continuously rotated around the vertical axis. (**b**) Charging voltage curve (C/5) including the evolution of a 2D (200) LFP and (200) FP peak showing the progressive FP formation and LFP disappearance.

Because of the micron-sized and bright X-ray beam, the diffraction rings that are observed by powder diffraction break up into individual spots, each representing an individual nanosized LiFePO_4_ grain (140 nm average particle size with no apparent preferential shape, X-ray diffraction (XRD) and scanning electron microscopy shown in Supplementary Fig. 1) in the battery electrode, as schematically shown in Fig. 1 (see also Supplementary Fig. 2 for a typical raw data 2D microbeam diffraction pattern). Here we refer to a single grain or particle as a single crystallite as opposed to particles that exist of a number of agglomerated crystallites. The high X-ray energy of 40 keV is transmitted by the coffee bag cells which comprise of a Li-metal negative electrode and a LiFePO_4_ (carbon-coated 140 nm crystallites) positive electrode. For the prepared LiFePO_4_ electrodes the micron-sized beam leads to typically tens of LiFePO_4_ grains that are in the diffraction conditions. Rotation of the cells over an angle *ω* perpendicular to the X-ray beam brings more grains in the diffraction condition and hence increases the chance to monitor transforming grains over time. In this way the phase transition behaviour of many individual grains (≈150 in the present experiment) is monitored as a function of time, while varying the electrochemical conditions from slow (C/50) up to fast rates (2C), where C/*n* denotes the rate at which a full charge or discharge takes *n* hours. The intensity of a single diffraction spot on a specific diffraction ring (defined by the diffraction angle *θ*) and position on the diffraction ring (defined by the azimuth angle *η*) represents the phase volume (of the phase corresponding that specific *θ*) in an individual LiFePO_4_ grain. Therefore, measuring the 2D diffraction patterns as shown in Fig. 1 allows to monitor the intensity of many individual LiFePO_4_ grains during operando (dis)charging, providing direct information on the time evolution and shape of phase domains in individual LiFePO_4_ grains. This is illustrated in Fig. 1 showing the evolution of the (200) reflection of a single LFP grain and a single FP grain during charge, where the gradual disappearance of the (200) LFP and appearance of the (200) FP diffraction peaks reflect the first-order phase transition (in a second order transition the peak would shift gradually in position).

### Transformation times of individual grains

**Figure 2:**
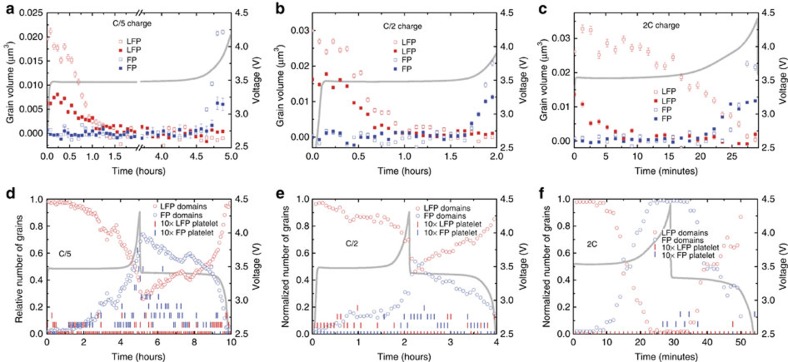
Time evolution of LiFePO_4_ and FePO_4_ grains. (**a**–**c**) Volume evolution of two LFP and FP domains during C/5, C/2 and 2C (dis)charge rate including the corresponding voltage curves. (**d**–**f**) Evolution of the relative number of LFP and FP grains, and the fraction 10*x* of which are streaks during different (dis)charge rates including the corresponding voltage curves. The phase transformation for C/2 (**e**) and 2C (**f**) appear delayed and ahead of the (dis)charge curves, respectively. This is most likely the result of a difference in activity in the small area that is probed by the microbeam as compared to the complete electrode.

Counting the number of FP and LFP reflections from individual grains during cycling, shown in Fig. 2d–f, reflects the reversible transformation from LFP to FP and back for the approximately 150 grains that were monitored. The intensity evolution of the reflections reveals for the first time the transformation of individual grains in electrode materials during cycling, as shown for two FP and two LFP grains in Fig. 2a–c for cycling rates of C/5 up to 2C. Averaging the transformation time of the ∼150 grains, the distribution of which is shown in the Supplementary Fig. 3), results in the average transformation time. At a relatively slow cycling rate of C/5 the average transformation time of individual crystallites is found to be *<Δt>*_LFP_=66 min and *<Δt>*_FP_=37 min (see Supplementary Fig. 3). This relatively slow transformation of individual LiFePO_4_ crystallites opposes the fast transformation suggested by the currently most widely accepted particle-by-particle transformation^14^,^17^,^18^,^19^,^20^.

The transformation time for 100–200 nm particles has been suggested to be of the order of a minute at these relatively low (dis)charge rates, assuming a surface reaction limited process^14^. The consequence of the much larger transformation times observed at present is that also the fraction of actively transforming particles (on average 22% during charge and 12% during discharge) is much larger than expected based on the particle-by-particle transformation^14^. Interestingly, Fig. 2a–c show that increasing the (dis)charge rate also increases the transformation rate. At C/2 the average transformation time decreases slightly to *<Δt>*_LFP_=50 min and *<Δt>*_FP_=31 min, whereas it decreases significantly at 2C to *<Δt>*_LFP_=17.6 min and *<Δt>*_FP_=7.8 min (see Supplementary Fig. 3). Although the transformation time decreases, the shorter (dis)charge time results in an increasing actively transforming fraction for increasing (dis)charge rates in qualitative agreement with recent findings^14^, albeit having much larger values, amounting almost 60% during 2C charge and 26% during 2C discharge. This shows that the increased current at higher (dis)charge rates is realized by both increasing the transformation rate of individual grains and by increasing the actively transforming fraction.

Figure 2a–c show that, in contrast to previous suggestions^33^,^34^, the transformation rate during discharge from Li-poor heterosite to Li-rich triphilyte appears to be larger. Whether this will result in a faster discharge also depends in the asymmetry of the charge transfer and of the Li-ion transport through the electrolyte. The cycling rate-dependent transformation times may be explained by either a charge transfer dependence, that is, Butler–Volmer, or a different transformation mechanism when the rate increases, allowing for much higher local current densities and larger actively transforming fractions, as will be discussed below.

### The phase transformation mechanism at slow cycling rates

**Figure 3:**
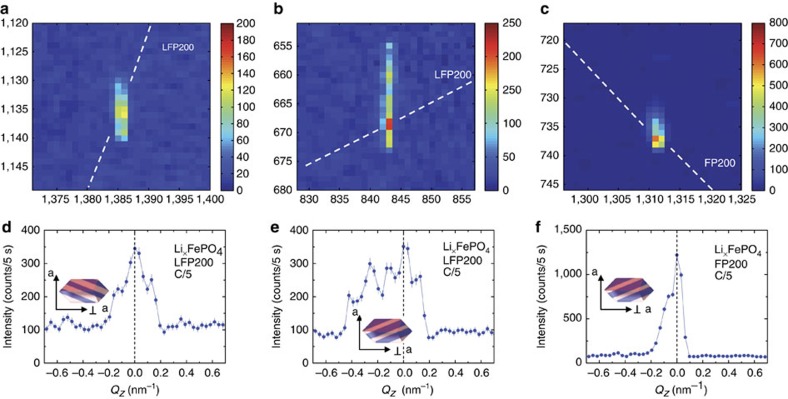
Phase transformation mechanism at slow cycling rates. (**a**–**c**) For the slow C/5 charge rate vertical streaks are observed for the (200) reflections of both the transforming LFP (**a**,**b**) and forming FP (**c**) phases. The horizontal and vertical axis of the 2D diffraction patterns are the detector positions. The dashed line indicates the (200) powder diffraction ring. (**d**–**f**) Distribution in intensity of the reflections (**a**–**c**) as a function of the vertical wave vector transfer *ΔQ*_*z*_ with respect to the central value. The position of the streak on the (200) diffraction ring, expressed in the azimuth angle *η*, defines the relative orientation of the normal of the platelet-shaped domain with respect to the a-axis, and the broadening in the *z*-direction the thickness of the platelet domains for the streaks shown leading to: (**a**) *η*=70°, *Δ*=20 nm, (**b**) *η*=209°, *Δ*=10 nm, (c) *η*=132°, *Δ*=43 nm. This leads to the shown proposed internal two-phase FP/LFP morphology.

A surprising observation is that a significant fraction of the (200) LFP and (200) FP reflections during C/5 (dis)charge appear as streaks as illustrated by the reflections in Fig. 3a–c, rather than symmetric peaks as shown in Fig. 1. The considerable one-dimensional broadening (the instrumental resolution is ∼2–3 pixels) infers platelet-shaped domains of both FP and LFP, where the plate thickness is inversely proportional to the length of the streaks^35^ (see Supplementary Note 1). For the observed streaks this results in a distribution in the plate thickness with an average value of ∼37 nm at C/5 (see Supplementary Fig. 4). This is considerably smaller than the pristine particle size (140 nm). The dimensions in the plate direction must be closer to the pristine particle size given the absence of broadening in these directions. It should be realized that in the present measurements streaks are only observable for the platelet domains that are aligned with the rotation axis *ω* (ref. 35), and as a consequence the number of platelet domains, shown in Fig. 2d–f, is significantly underestimated.

The position of the streaks on the (200) diffraction ring (characterized by the azimuth angle *η* indicated in Fig. 1) in combination with the streak orientation allows us to determine the crystallographic orientation of the platelet-shaped domains in the LiFePO_4_ grains (see Supplementary Note 2). At C/5 the distribution in *η* for all observed streaks (Supplementary Fig. 4) exhibits broad maxima from *η*≈60°–135° and *η*≈240°–300°, indicating a range of preferred orientations of the platelet domains having their normal under an angle in the range of 0°–60° relative to the crystallographic *a*-axis.

Although the present results only provide the relative crystallographic orientation of the plane normal with respect to the *a*-axis, the emerging phase morphology, as sketched in Fig. 3d–f, is consistent with a mixture of the two morphologies predicted by Cogswell *et al*.^12^ by the minimization of the coherency strain between the coexisting phases, (1) the striped morphology with interfaces in the (101) direction (consistent with *η*≈60°, 120°, 240° and 300°) obtained for coherent FP/LFP interfaces by and (2) the striped morphology with interfaces in the (100) direction (consistent with *η*≈90° and 270°) because of the loss of coherence in the (001) direction. This indicates that in these 140 nm particles a distribution exists of FP/LFP interfaces ranging from coherent to partially coherent, to complete loss of coherence in the (001) direction. Also the length scale for the platelet domain separation, or average platelet domain thickness, 37 nm at C/5 (Supplementary Fig. 4) is in good agreement with phase-field modelling^12^ and TEM observations^29^,^36^. The complete loss of coherence potentially leads to cracks in the (100) direction which is consistent with observations in micron sized LiFePO_4_ grains^29^, indicating that this microscopic domain behaviour plays a role even in these relatively small particle sizes most likely decreasing their cycle life.

Coexisting phase morphologies are predicted for single grains^12^ and are supressed by taking into account the coherency strain in multiple grain phase-field models that allow inter-particle charge transport^25^. It should be noted that phase-field modelling does predict mosaic instabilities, causing a phase coexistence to occur locally^37^. However throughout the electrodes coexisting phases are penalised by the coherent strain energy and this leads to the overall prediction of a mosaic phase transformation^25^. However, the streaked reflections unambiguously represent particles having an internal structure with coexisting FP and LFP phases during C/5 (dis)charge. In combination with the slow transformation rate of individual particles the internal phase structure observed at present this exposes a very different picture from the particle-by-particle, or mosaic, phase transformation mechanism, which is generally assumed to occur in these relatively small LiFePO_4_ grains at low (dis)charge rates^14^,^17^,^18^,^19^,^20^.

At first glance, it appears surprising that the internal phase structure sketched in Fig. 3d–f, resulting from the appearance of streaks, is not observed by previous studies. However, only few of these studies operate under realistic *in-situ* conditions and powder diffraction is much less sensitive to the one-dimensional character of the domains. This is illustrated by the powder X-ray data obtained from the same electrodes that exhibit streaks under microbeam conditions, which does not show obvious broadening under powder diffraction conditions (using a larger beam)^9^, as shown in in the Supplementary Fig. 5. This is consistent with the absence of a significant peak broadening in earlier XRD studies that initiated the particle-by-particle transformation picture in LiFePO_4_ (ref. 17). The limited length scale and the anisotropic nature of the domains occurring in only a fraction of the grains (less at lower (dis)charge rates) also appears to mask the observation of the internal domain structure for the recently reported X-ray microscopy^14^,^20^ and TEM studies^18^,^19^. The present observations of the internal platelet domain structure in individual LiFePO_4_ particles has been made possible by micro-focus hard X-ray diffraction because a statistical ensemble of crystallites could be monitored under *in-situ* conditions.

To check the stability of the internal domain structure at even lower rates, a partial C/50 charge was conducted, also resulting in the appearance of streaked reflections as shown in Supplementary Fig. 6. At C/50 both the average platelet thickness and the distribution in the azimuth angle *η* are comparable to C/5 (dis)charge rate, demonstrating a comparable internal platelet domain structure, see Supplementary Figs 4 and 6. The appearance of streaks during C/50 charging indicates that, even at these low rates, the internal domain structure does not transform to what should be expected to be the lower energy configuration for a multi-particle ensemble where each particle has a single phase^12^,^21^,^25^,^38^, avoiding the coherency strains associated with the internal interfaces^12^,^21^,^25^,^37^. Possibly, this is related to the poor kinetics of the interfaces related to the associated elastic energy^31^.

### The phase transformation mechanism at fast cycling rates

**Figure 4:**
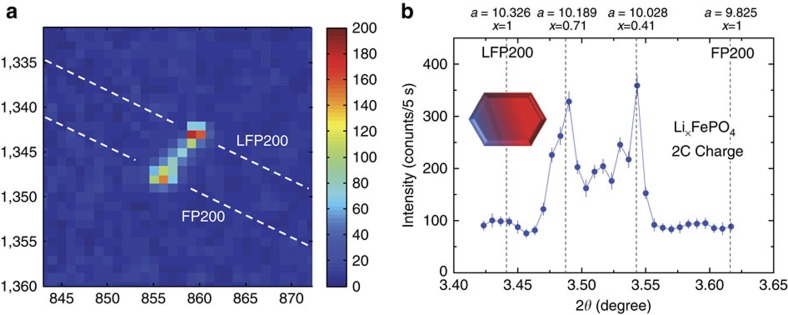
Phase transformation mechanism at fast cycling rates. (**a**) (200) reflection at 2C charging rate showing coexistence of the LFP and FP phases within a single grain. The dashed lines indicate the powder rings for the (200) reflections of the LFP and FP phases, indicated as LFP200 and FP200, respectively. (**b**) The corresponding line scan is shown as function of the scattering angle 2*θ*. The maximum intensity is observed at scattering angles corresponding to a=10.180 Å (*x*=0.71) for the LFP phase and a=10.028 Å (*x*=0.42) for the FP phase. For both phases the *a*-axis is closely aligned indicating a diffuse coherent interface.

Drastic changes set in when the cycling rate is increased from C/50 and C/5 to C/2 and 2C causing a decrease in the relative number of streaked reflections and a gradual narrowing of the streaks. This translates into a decrease in number of platelet domains, as illustrated by the platelet domain statistics in Fig. 2d–f, and an average increase in platelet domain thickness (see Supplementary Fig. 4) upon increasing the current. Fig. 4 shows the striking appearance of a double peaked reflection at 2C charging rate. It provides direct evidence of a diffuse interface, and phase compositions far from their equilibrium values, within a single grain. The LFP (200) and FP (200) reflections can still be distinguished in Fig. 4, resulting in strongly deviating *a*-axis lattice parameters (compared to the end-member values), which implies Li-compositions far away from the equilibrium compositions. The appreciable intensity between the two reflections indicates a continuous distribution of intermediate compositions, reflecting a diffuse interface over the full-length scale of the 140 nm grain. This provides unique insight in the internal phase structure in a single grain at fast cycling, indicating that the recently observed unstable intermediate compositions^9^,^10^,^39^,^40^ can occur within a single grain, as proposed by Zhang *et al*.^9^, Liu *et al*.^10^ and Orikasa *et al*.^39^,^40^ and as predicted by phase-field modelling^12^.

The disappearance of the well-defined anisotropic internal domain structure when the rate increases from C/5 up to 2C in (Supplementary Fig. 4d–f) goes along with a decrease in the average transformation time (Supplementary Fig. 3). This is indicates that the platelet-shaped domain structure, with well-defined phase boundaries, may be the origin of the relatively long transformation times. In contrast, the more diffuse interfaces, first experimentally suggested by powder diffraction^10^ and at present observed directly, driven by higher overpotentials as predicted by phase-field modelling^11^, allow for a much faster transformation and therefore higher current densities, explaining the high-rate performance of LiFePO_4_.

## Discussion

**Figure 5:**
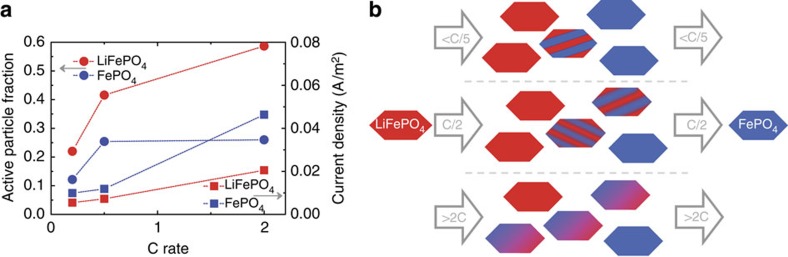
The cycling rate-dependent phase transformation mechanism. (**a**) Active fraction and current density of the particles resulting from the average transformation times. (**b**): Sketch of the rate-dependent transformation upon charge as follows from the microbeam diffraction experiments (similar upon discharge only with a smaller active particle fraction, see (**a**)).

The present microbeam X-ray diffraction results reveal for the first time the phase transformation in a large number (≈150) of individual electrode particles under realistic operando conditions, bringing forward a new fundamental insight in the rate-dependent phase transformations taking place in LiFePO_4_ electrodes. As schematically shown in Fig. 5b, at low (dis)charge rates the streaked reflections reveal that the transformation occurs concurrently in a large fraction of the grains and that it proceeds via the phase coexistence of thin platelet FP and LFP domains in individual grains. The distribution in relative orientations of the interface with respect to the *a*-axis, between the (101) and (100) planes, indicates the presence of coherent interfaces, as well interfaces with partial or complete loss in coherency in the (001) direction. In combination with the slow transformation rate of individual grains, reported for the first time, gives a very different mechanistic picture compared to the particle-by-particle, or mosaic, transformation that is commonly assumed to occur at relatively low (dis)charge rates^14^,^17^,^18^,^19^,^20^.

The results indicate that the reaction kinetics observed here at low rates, is much slower than previously assumed^17^ and predicted^25^. Even at C/50 rates the internal platelet domain structure remains. The well-defined internal domain structure may be responsible for the observed slow transformation rates for individual grains at low (dis)charge rates, possibly due to pinning of the domain walls on defects created by the loss in coherency. Another explanation for the slow transformation kinetics may be a very slow charge transfer reaction. Bazant *et al*.^41^ reported an exchange current density of about 10^−4^ A m^−2^, smaller, but not very different from the order of magnitude reported at C/5 in Fig. 5a. Such slow charge transfer kinetics would effectively freeze the internal domain structure. The presence of (100) interfaces indicates the loss in interface coherency, potentially leading to mechanical failure that reduces the cycle life, even for these relatively small 140 nm LiFePO_4_ grains. Increasing the (dis)charge rate suppresses the formation of sharp interfaces, as demonstrated by the vanishing streaked reflections, which represent the platelet-shaped domains. The broad diffuse interface with intermediate compositions, at present observed in single particles, is expected to reflect a larger domain wall mobility due to less concentrated coherency strains making pining on defects less likely, providing a possible explanation for the faster transformation times observed at present and the high intrinsic rate performance of LiFePO_4_. This would imply that the observed transformation rates of individual grains depends on the presence of defects, the creation of which should be avoided by fast cycling and or by tailoring the particle size and shape to minimize coherency strain effects, thereby preventing the formation of defects. On the other hand if the charge transfer kinetics is rate limiting, this may potentially be improved by surface coatings that allow faster charge transfer.

Measuring the transformation process of individual LiFePO_4_ particles allows for the first time quantification of local current densities in Li-ion battery electrodes under realistic (dis)charge conditions, as shown in Fig. 5a. The present values are significantly larger compared with what is typically obtained when assuming that all electrode active material participates^42^ and significantly smaller when assuming a particle-by-particle dominated mechanism^14^. The (dis)charge rate dependence of the local current density (shown in Fig. 5a) which was derived from the average transformation times of individual grains is a crucial parameter for electrode design that can help to prevent the occurrence of hotspots and fractures in electrodes^15^,^16^. The increase in the local current density is a direct consequence of the faster transformation of individual particles upon increasing the (dis)charge rate. In combination with the increasing fraction of actively transforming particles, in qualitative agreement with recent findings^14^, the faster transformation mechanism is responsible for supplying the larger currents when increasing the (dis)charge rates.

The dynamically induced diffuse interfaces at high (dis)charge rates means that the build-up of local of the coherent lattice strain at the sharp phase boundaries is avoided. This is expected to prevent the formation of cracks at high (dis)charge rates, counter intuitively predicting that the LiFePO_4_ material should show a longer cycle life time when cycled at higher rates. Possibly, for LiFePO_4_ particles, a reduced size of the order of the observed platelet domains, will not permit the existence of well-defined phase boundaries, thereby preventing potential crack forming and extending the cycle life also at low (dis)charge rates.

Given the general nature of the phase transition behaviour observed here, an interesting future question is how these findings will translate towards other phase separating insertion materials, a group of materials that are highly relevant for energy storage. The results illustrate the unique abilities of microbeam diffraction revealing the fundamental phase transition processes in electrodes under realistic operando conditions and providing guidance for future battery design.

## Methods

### Sample preparation

he starting material was carbon-coated LiFePO_4_ from Phostech with an average particle size of 140 nm. LiFePO_4_ cathodes were prepared through mixing a slurry of LiFePO_4_, Carbon Black (Super P), PVDF, (polyvinylidene fluoride, Solvay) in NMP (N-methylpyrrolidone, with a mass ratio of the active material (LiFePO_4_), carbon black (SuperP) and binder (PVDF) of 80:10:10. The final slurry was casted on carbon-coated aluminium current collectors by doctor blading. The coatings were dried on a heater plate under air at ∼155 °C overnight followed by drying under vacuum at around 60 °C for more than 24 h. The resulting coatings were pressed using a roller hand press to enhance the electronic contact. Finally, the electrodes are dried for at least 3 h under vacuum at 100 °C.

### Battery preparing and testing

The electrodes were assembled in ‘coffee bag’-type cells all assembled under argon atmosphere (<0.1 p.p.m. O_2_/H_2_O). The electrodes were separated by glass microfibre filters (Whatman) with a few droplets of 1 mol l^−1^ LiPF_6_ (EC:DMC 1:1, Novolyte) electrolyte. All the electrochemical tests were performed galvanostatically within a voltage window of 4.3 and 2.5 V versus Li/Li+ using an Autolab PGSTAT302N potentiostat/galvanostat.

### Operando synchrotron X-ray diffraction

The *in-situ* synchrotron XRD experiments were performed at the ID11 beam line of the European Synchrotron Radiation Facility (ESRF Grenoble, France). Fig. 1 shows the experiment setup. A monochromatic X-ray with an energy of 40 keV (wavelength 0.30996 Å) and a beam size of 1.7 μm was used to illuminate the coffee bag cell. The grains that fulfilled the Bragg condition generated a diffraction spot on the 2D FReLoN2k coupled-charges device detector placed behind the sample. The grain volume of individual grains was determined as described in Supplementary Note 3. During exposure the sample was continuously rotated around the axis perpendicular to the X-ray beam over an angular range 0.5° with an exposure time of 10 s (C/50, C/5, C/2) or 5 s (2C). By collecting several subsequent angular exposures a total angular range of 6° (2C, C/50) or 12° (C/2, C/5) was covered, resulting in a time resolution for individual measurements cycles of 1 min (2C), 4 min (C/2, C/5) and 2 min (C/50). The instrument parameter were determined using CeO_2_ as calibrant.

## Additional information

**How to cite this article:** Zhang, X. *et al*. Direct view on the phase evolution in individual LiFePO_4_ nanoparticles during Li-ion battery cycling. *Nat. Commun.* 6:8333 doi: 10.1038/ncomms9333 (2015).

## Supplementary Material

Supplementary Information Supplementary Figures 1-6, Supplementary Notes 1-3 and Supplementary References
