# Supplementary material for: Direct view on the phase evolution in individual LiFePO4 nanoparticles during Li-ion battery cycling
Source: Nat Commun. 2015 Sep 23;6:8333. doi: 10.1038/ncomms9333 (PMC4597332; doi:10.1038/ncomms9333)
Supplement: Supplementary Information — Supplementary Figures 1-6, Supplementary Notes 1-3 and Supplementary References [file ncomms9333-s1.pdf]

Supplementary Figures

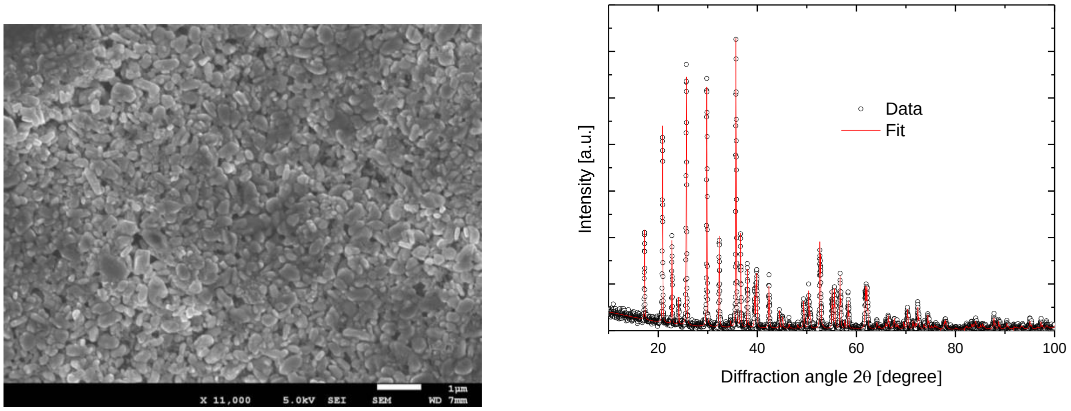

Supplementary Figure 1. *Left: SEM picture of the pristine $LiFePO_4$ material with an average particle size of 140 nm. Right: XRD (Cu-K_α) of the pristine $LiFePO_4$ material including fit (orthorhombic Pnma space group) yielding lattice parameters a= 10.329 Å, b=6.007 Å and c=4.691 Å.*

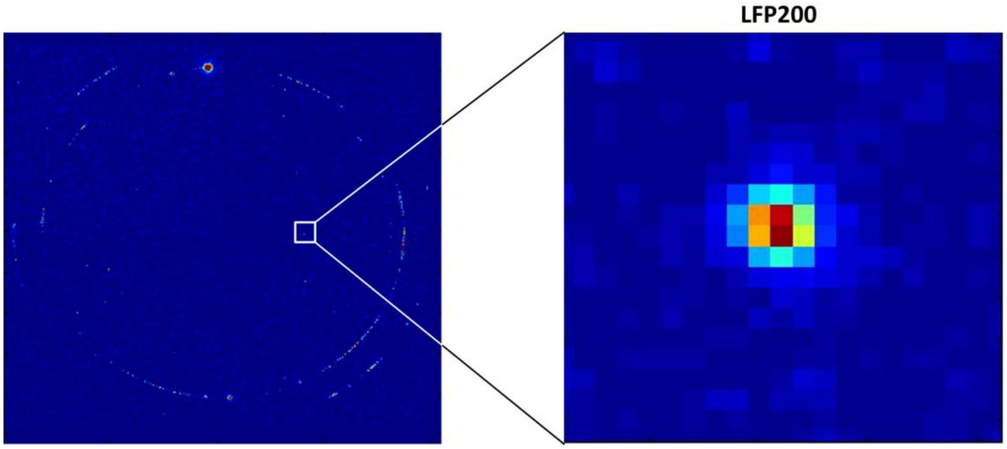

Supplementary Figure 2. *Example of a 2D XRD micro-beam pattern with an enlarged picture of a single (200) reflection of the $LiFePO_4$ phase, representing a single grain in the electrode.*

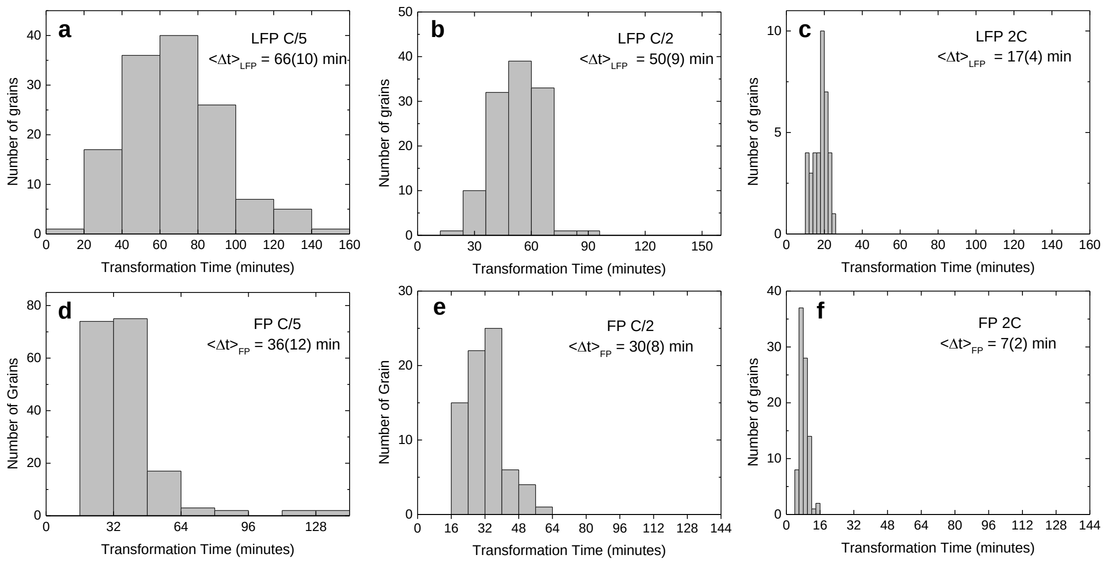

Supplementary Figure 3. *Distribution of grain transformation times with the average transformation time <Δt> for individual LFP and FP grains at different cycling rates.*

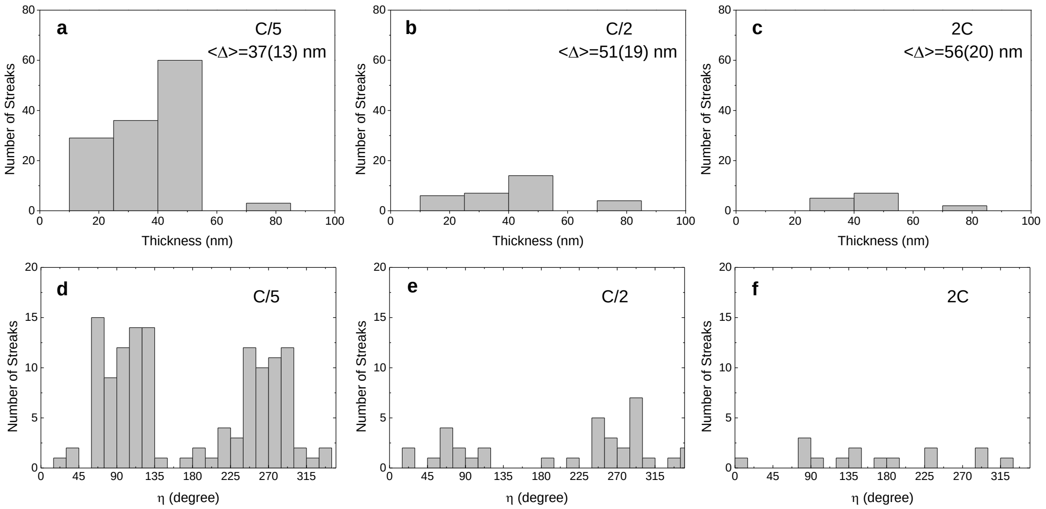

Supplementary Figure 4. *(a-c) Distribution of the platelet thickness deduced from observed streak length for different electrochemical (dis)charge rates, where <Δ> represents the average platelet thickness. (d-f) Distribution of the azimuth angle η of the streaks on the diffraction rings for different electrochemical (dis)charge rates.*

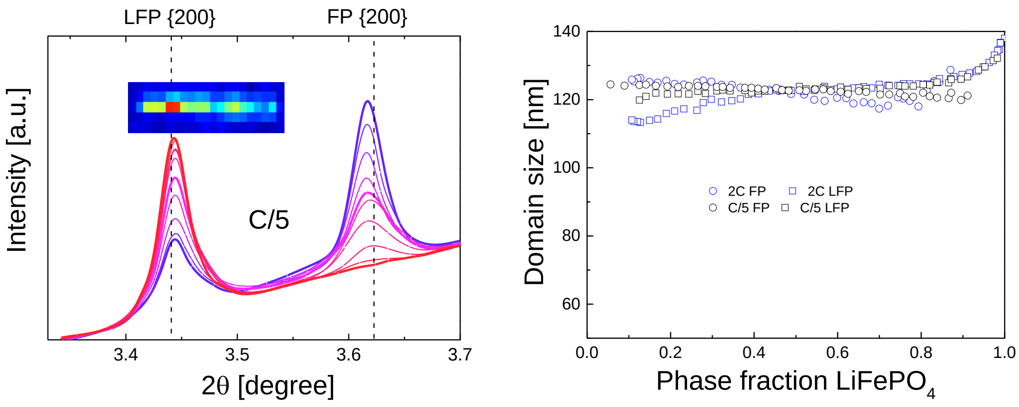

***Supplementary Figure 5**. Left: In-situ powder X-ray diffraction during C/5 charging subsequent after the micro beam diffraction experiment (same cell, same geometry, larger beam size, see ref. [1] for details). The streaks that appear during C/5 charging lead to an anisotropic broadening that is generally not observable, as it disappears in the background. Right: The evolution of C/5 and 2C broadening [1].*

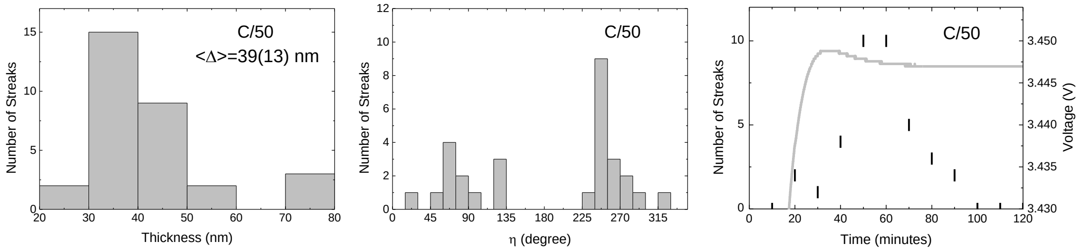

***Supplementary Figure 6.** Left: Distribution of thickness of the platelets during the partial C/50 charge. Middle: Distribution in the azimuth angle η during the partial C/50 charge. Right: Number of streaks versus time including the voltage evolution during the partial C/50 charge.*

Supplementary Notes

Supplementary Note 1.
Relation between the streak peak profile and the platelet domain thickness

The peak profile $I(\mathbf{Q})$ of the scattered intensity originating from a single particle at the Bragg condition $\mathbf{Q} = \mathbf{Q}_{hkl} + \mathbf{q}$ can be described by the form factor of the particle $F(\mathbf{q})$ as $I(\mathbf{Q}) \propto |F(\mathbf{q})|^2$. The form factor of a uniform rectangular bar (parallelepiped) corresponds to [2-4]:

$$F(\mathbf{q}) = L_x L_y L_z \left(\frac{sin(q_x L_x / 2)}{q_x L_x / 2} \right) \left(\frac{sin(q_y L_y / 2)}{q_y L_y / 2} \right) \left(\frac{sin(q_z L_z / 2)}{q_z L_z / 2} \right) \quad (1)$$

where L_x, L_y and L_z are the dimensions of the bar along the *x*, *y* and *z* directions. When the two in-plane dimensions can be assumed to be large only the plate thickness results in peak broadening. The scattered intensity then corresponds to:

$$I(\mathbf{Q}) \propto |F(\mathbf{q})|^2 \to (2\pi)^2 L_x L_y L_z^2 \delta(q_x)\delta(q_y) \left(\frac{sin(q_z L_z / 2)}{q_z L_z / 2} \right)^2 \quad (2)$$

where the plate normal is along the *z* direction. The full width at half maximum (FWHM) amounts to $\Delta q_z \approx 0.886 \times 2\pi / L_z$.

Supplementary Note 2.
Reflection condition for streaks and orientation of the plate normal with respect to the crystalline a-axis

For a scattering angle 2θ and an azimuth angle η on the 2D detector the **Q** vector corresponds to $\mathbf{Q} = (Q_x, Q_y, Q_z) = (-\sin\theta, -\cos(\theta)\sin(\eta), \cos(\theta)\cos(\eta))$ in laboratory coordinates. For a given rotation angle $\Delta\omega$ around the vertical axis the rotated coordinates are given by:

$$\mathbf{Q}' = (Q_x \cos(\Delta\omega) - Q_y \sin(\Delta\omega), Q_x \sin(\Delta\omega) + Q_y \cos(\Delta\omega), Q_z) \quad (3)$$

As the form factor is sharply peaked in reciprocal space, we only expect to observe streaks, originating from nanoscale plate structures, when Q_z is unaffected by the continuous sample rotation of 0.5° during exposure. This is only the case when the plate normal is aligned along the *z* direction. Otherwise, the plate will quickly rotate out of the reflection condition. The measured peak intensity after exposure at a constant rotation speed corresponds to:

$$I = \int_{\omega}^{\omega+\Delta\omega} I(\mathbf{Q}) d\omega' \quad (4)$$

For randomly plates the effective time the particle is in reflection is short, while for aligned particles streaks will be visible.

For observed (200) reflections of the LFP and FP phases the **Q** vector corresponds to $\mathbf{Q} = \mathbf{Q}_{200} = (-\sin\theta, -\cos(\theta)\sin(\eta), \cos(\theta)\cos(\eta))$, where 2θ is the scattering angle and an

azimuth angle η on the 2D detector. The orientation of **Q** is equal to the reciprocal-space **a*** axis, which for an orthorhombic lattice is aligned to the real-space **a** axis ($\mathbf{Q}_{200}$||**a***||**a**). For our current experimental conditions streaks are only observed when the plate normal **n** is oriented vertical (**n**||**z**). As the scattering angle 2θ of the (200) reflections for and are 3.44° (LFP) and 3.62° (FP), we find $\mathbf{Q}_{200} \approx (0, -\sin(\eta), \cos(\eta))$. This means that the azimuth angle η is about equal to the angle between the plate normal and the **a** axis of the LFP and FP phases when the (200) reflection is analyzed. For reflections on top (η = 0°) and bottom (η = 180°) of the diffraction ring the plate normal is aligned with the **a** axis. For reflections at the left and right extremes on the diffraction ring (η = 90° and η = 270°) the plate normal is perpendicular to the **a** axis.

Supplementary Note 3.
Determination of the grain volume

For diffraction spots the integrated intensity I_g is directly related to the grain volume V_g [5,6] by:

$$I_g = \Phi_0 r_0^{\,2} \frac{\lambda^3 |F_{hkl}|^2 V_g}{\Delta\omega |\sin\eta| v^2} L_g P T_r \exp(-2M) \tag{5}$$

where Φ_0 is the incident flux of photons, F_{hkl} is the structure factor of the (*hkl*) reflection, λ is the photon wavelength, $\Delta\omega$ is the angular range over which the grain is rotated, *v* is the volume of the unit cell, *P* is the polarization factor, and T_r is the transmission factor. The Lorentz factor of the grain is given by $L_g = 1/\sin(2\theta)$, where 2θ is the scattering angle, r_0 is the Thomson scattering length and exp(-2*M*) is the Debye-Waller factor.

For a powder diffraction experiment, the integrated intensity I_p of a diffraction ring of a polycrystalline material with randomly oriented grains is given by:

$$I_p = \Phi_0 r_0^{\,2} \frac{m_{hkl} \lambda^3 |F_{hkl}|^2 V}{v^2} L_p P T_r \exp(-2M) \tag{6}$$

where m_{hkl} is the multiplicity factor of the ring and *V* is the volume of the diffracting phase, and $L_p = 1/\{4\sin(\theta)\}$ is the Lorentz factor for an integrated powder ring. The volume of the diffraction phase is equal to $V = f\, V_{gauge}$, where *f* is the volume fraction of the diffraction phase and V_{gauge} is the gauge volume, which is defined by the beam size and the thickness of the sample. The layer thickness was 10.8(4) μm. With a phase fraction of 80% for the active phase and a porosity of 34% the phase fraction is $f = 0.80 \times 0.66 \times f_{LFP/FP} = 0.53 \times f_{LFP/FP}$.

The volume of an individual grain is calculated from the integrated intensity of an individual (*hkl*) reflection normalized by the powder intensity of the ring in which the reflection from the individual grain appeared:

$$V_g = \frac{1}{2} m_{hkl} \Delta\omega \,|\sin\eta|\, \cos(\theta) f V_{gauge} \left(\frac{I_g}{I_p} \right) \tag{7}$$

In addition we show that the chance of overlap of reflections is extremely small. Considering the illuminated sample volume and the particle size we calculate that the X-ray beam illuminates about 1000 particles. For a single exposure with a continuous rotation over an angle of 0.5 degree only a fraction of 0.5/180 = 1/360 of the total solid angle (4π) is probed. With a multiplicity of m_{hkl} = 2 for the {200} LFP/FP reflection only 2xm_{hkl}x1000/360 ≈ 10 grains are expected to fulfil the Bragg condition. This means that the reflections have an average spacing of about 36 degrees along the diffraction ring. With a mosaicity below 0.25 degree the chance of overlap is extremely small.

Supplementary References

[1] X. Zhang, M. van Hulzen, D.P. Singh, A. Brownrigg, J.P. Wright, N.H. van Dijk and M. Wagemaker, Nano Lett. 14 (2014) 2279-2285.

[2] D.S. Sivia, *Elementary scattering theory for X-ray and neutron users*, Oxford University Press (2011) Oxford.

[3] L.A. Feigin and D.I. Svergun, *Structure analysis by small-angle X-ray and neutron scattering*, Plenum Press (1987) New York.

[4] Y. Takano, K.N. Liou, P. Yang, J. Quantitative Spectroscopy Radiative Transfer 113 (2012) 1836–1843.

[5] J. Als-Nielsen and D. McMorrow, *Elements of Modern X-ray Physics*, Wiley, West Sussex (2001).

[6] B. E. Warren, *X-ray Diffraction*, Dover Publications, New York, (1990).
